# Male Sex and Obesity are Associated with Sarcopenia in Patients with Rheumatoid Arthritis in the 2008–2011 Korea National Health and Nutrition Examination Survey

**DOI:** 10.3390/jcm15083148

**Published:** 2026-04-20

**Authors:** Yoon-Jeong Oh, Chang-Nam Son

**Affiliations:** Division of Rheumatology, Department of Internal Medicine, Uijeongbu Eulji Medical Center, Eulji University School of Medicine, Uijeongbu 11749, Republic of Korea; yjgark640@gmail.com

**Keywords:** rheumatoid arthritis, sarcopenia, obesity, sex

## Abstract

**Background/Objectives**: The objective of this investigation was to assess the prevalence of sarcopenia and identify its related risk factors in patients with rheumatoid arthritis (RA) using data from the nationally representative 2008–2011 Korea National Health and Nutrition Examination Survey (KNHANES). **Methods**: We analyzed data from the 2008–2011 KNHANES to identify the factors associated with sarcopenia in patients with RA. Sarcopenia was defined as the ratio of appendicular skeletal muscle mass to total body weight (multiplied by 100), with cut-offs of <29.0% for men and <22.9% for women. To identify the specific factors independently associated with sarcopenia, a multivariate logistic regression model was employed, accounting for sample weights and the complex survey design. **Results**: Among 238 patients with RA included in the analysis, 44 (weighted prevalence: 22.7%) had sarcopenia. The sarcopenia group had a higher proportion of males (55.0% vs. 15.3%, *p* < 0.001), body mass index (BMI) (26.1 vs. 23.3 kg/m^2^, *p* < 0.001), and waist circumference (86.9 vs. 79.0 cm, *p* < 0.001) than the non-sarcopenia group. After adjustment for potential confounders, including age, sex, obesity, physical activity, and daily protein intake, male sex (odds ratio [OR]: 4.17; 95% confidence interval [CI]: 1.48–11.77, *p* = 0.007) and obesity (OR: 3.06; 95% CI: 1.16–8.07, *p* = 0.024) were independently associated with sarcopenia. In sex-specific analyses, low physical activity was significantly associated with sarcopenia only in male patients (OR: 13.00; 95% CI: 1.90–88.75, *p* = 0.012). **Conclusions**: Our findings indicate that being male and having a higher BMI are significant independent indicators of sarcopenia risk within the Korean RA population. This highlights their critical role in the development of sarcopenia among RA patients.

## 1. Introduction

Rheumatoid arthritis (RA) is a systemic, immune-mediated disorder characterized by chronic inflammation of the joints, leading to persistent synovitis, pain, and progressive joint destruction [1]. In addition to joint involvement, RA often leads to various systemic complications, such as fatigue, cardiovascular disease, and sarcopenia, all of which substantially impair functional ability and overall quality of life [2]. Chronic systemic inflammation in RA is a key mechanism that drives both musculoskeletal decline and cardiovascular risk [3]. Specifically, this persistent inflammatory state accelerates atherosclerosis and disrupts the balance of oxygen supply to the heart, which is central to the development of Type 2 myocardial infarction (T2MI) [4]. This highlights that RA should be managed as a complex systemic disorder with broad impact on multiple organs, rather than just a localized joint disease.

Sarcopenia is defined by a progressive reduction in skeletal muscle mass, muscle power, and physical performance [5]. Although sarcopenia is commonly associated with aging, it can develop secondary to chronic conditions, including RA, malignancy, or chronic systemic diseases, affecting the kidneys, liver, heart, or lungs [6,7]. Several studies suggest that although sarcopenia affects 5–13% of the general population aged 60–70 years, its prevalence in patients with RA may be >25% [8,9]. The high prevalence of sarcopenia in this population is associated with reduced physical activity due to pain and sustained systemic inflammation [5,10]. Proinflammatory cytokines, including tumor necrosis factor-alpha (TNF-α) and interleukin (IL)-6, accelerate muscle catabolism and impair repair mechanisms, resulting in a more rapid muscle loss than occurs with aging alone [10,11]. These inflammatory pathways may also interact with metabolic processes such as insulin resistance and adipokine dysregulation, further worsening clinical outcomes and potentially promoting sarcopenic obesity [12]. Consequently, patients with both RA and sarcopenia are at increased risk of physical disability, falls, fractures, and mortality, imposing a substantial burden on healthcare systems [10,13].

Recent observations indicate that muscle loss in RA does not follow a single pattern but is instead influenced by complex factors, including sex and obesity [14,15]. The interaction between hormonal regulation, adiposity, and inflammation may lead to distinct clinical outcomes between men and women. However, comprehensive studies are still needed to better understand how these factors contribute to RA-associated sarcopenia. In particular, despite its clinical significance, large-scale, population-based studies using standardized, weight-adjusted appendicular skeletal muscle mass (ASM) criteria validated in the Korean population are lacking. Most previous studies used small clinical cohorts or inconsistent definitions of sarcopenia, limiting the comparability and generalizability of their findings [5,16].

Therefore, this study sought to investigate the prevalence and determinants of sarcopenia among Korean adults with RA, based on data from the Korea National Health and Nutrition Examination Survey (KNHANES). We specifically examined how sex and body composition relate to sarcopenia risk. This focus may provide practical insights into the clinical burden of sarcopenia in patients with RA. A better understanding of these relationships may help clinicians identify high-risk individuals and develop targeted strategies to prevent and manage sarcopenia in this population.

## 2. Materials and Methods

### 2.1. Data Source and Study Design

This study utilized data from the 2008–2011 cycles of the KNHANES, a nationally representative survey conducted to assess the health and nutritional status of the Korean population [17]. Among 16,378 participants, 238 adults (≥19 years) with RA were included in the analysis. RA was defined as self-reported physician-diagnosed RA or current treatment for RA based on the KNHANES health interview. In KNHANES, disease status is assessed through standardized health interviews, in which participants are asked whether they have ever been diagnosed with RA by a physician or are currently receiving treatment for RA. This study was approved by the Institutional Review Board of the Uijeongbu Eulji Medical Center (UEMC 2025-08-002-001), and conducted in accordance with the Declaration of Helsinki.

### 2.2. Definition of Sarcopenia and BMI Categories

In the KNHANES, ASM was assessed via dual-energy X-ray absorptiometry (QDR 4500A; Hologic Inc., Bedford, MA, USA) [18]. ASM was defined as the sum of lean soft tissue mass in the upper and lower extremities, excluding fat and bone components, and is considered a surrogate measure of skeletal muscle mass [19]. Sarcopenia was operationally defined using weight-adjusted ASM (ASM/weight × 100), which accounts for differences in body size and composition. Sex-specific cut-off values (<29% for men and <22.9% for women) were applied based on Korean population reference data [9,20]. This definition has been widely applied in previous KNHANES-based epidemiological studies and is considered appropriate for reflecting body composition in Asian populations [21,22,23]. Body mass index (BMI) was categorized based on the Korean Society for the Study of Obesity recommendations and divided into the following categories: underweight (<18.5 kg/m^2^), normal weight (18.5–22.9 kg/m^2^), overweight (23.0–24.9 kg/m^2^), and obese (≥25.0 kg/m^2^) [24].

### 2.3. Health and Dietary Behavioral Characteristics

Health-related behaviors and clinical variables were assessed using standardized protocols from the KNHANES health interview and examination surveys conducted between 2008 and 2011. Information on health-related behaviors (e.g., smoking, alcohol consumption, and physical activity) was collected via standardized self-reported questionnaires administered by trained interviewers, while clinical measurements (e.g., blood pressure, laboratory values, and physical characteristics of the participants) were obtained through direct physical examinations and laboratory analyses. Alcohol consumption was defined as drinking at least once a month over the past year. Current smoking status was defined as smoking daily or occasionally during the survey. Physical activity was assessed using the Korean version of the International Physical Activity Questionnaire short form. Based on the World Health Organization (WHO) guidelines on physical activity for health, participants were classified as meeting recommendations if they engaged in ≥150 min per week of moderate-intensity activity or ≥75 min per week of vigorous-intensity activity [25]. Those who did not meet these criteria were categorized into the physically inactive group. Hypertension (HTN) was identified based on a systolic blood pressure ≥ 140 mmHg or a diastolic blood pressure ≥ 90 mmHg. Additionally, participants with a documented physician diagnosis or those undergoing antihypertensive therapy were also included in this category. Diabetes mellitus (DM) was defined as a fasting plasma glucose level ≥ 126 mg/dL, a physician diagnosis, or current treatment for diabetes. Hyperlipidemia was defined as a fasting total cholesterol level ≥ 240 mg/dL, a physician diagnosis, or current use of lipid-lowering medications. Only participants who had fasted for at least 8 h prior to blood sampling were included for the laboratory-based definition to improve data accuracy. Osteoarthritis (OA) and depression were defined based on physician diagnosis, current treatment, or current disease status. Osteoporosis was defined according to bone mineral density measurements, with a T-score ≤ −2.5 at the total femur, femoral neck, or lumbar spine. We assessed health-related quality of life using the EuroQol-5 Dimension (EQ-5D) index, a standardized tool that encompasses five dimensions [26]. These domains include physical mobility and self-care, as well as the performance of daily activities, the presence of pain or discomfort, and mental health status regarding anxiety or depression [26]. The resulting index score ranges from 0 to 1, with higher values indicating a better health status. We used the Korean-specific valuation set to calculate the final index score [27].

### 2.4. Statistical Analysis

All statistical analyses were performed using SPSS version 23.0, accounting for the complex survey design of the KNHANES by incorporating sampling weights, strata, and clusters. The resulting weighted estimates are representative of the Korean population and reflect population-level values rather than raw sample data. Continuous variables are expressed as weighted means with 95% confidence intervals (CIs), while categorical variables are presented as weighted proportions. Group comparisons were performed using the complex samples general linear model and the Rao–Scott adjusted chi-square test. Independent factors associated with sarcopenia were examined using multivariate logistic regression analyses, with variables selected based on clinical relevance and univariate significance. This model further evaluated the association of sex and BMI, adjusting for age and other potential confounders. The results are presented as adjusted odds ratios (ORs) with 95% CIs. Weighted proportions and their 95% CIs were calculated using the Taylor series linearization method within the complex samples module of SPSS. All statistical tests were two-tailed, and a *p*-value < 0.05 was considered statistically significant.

## 3. Results

### 3.1. Comparison of Baseline Characteristics Between the Non-Sarcopenia and Sarcopenia Groups

A total of 238 patients with RA were included in the analysis, of whom 44 (weighted prevalence, 22.7%) were classified as having sarcopenia. Table 1 summarizes the baseline characteristics of the study population according to sarcopenia status. Compared with individuals without sarcopenia, those with sarcopenia were more likely to be male (55.0% vs. 15.3%, *p* < 0.001) and had significantly higher BMI (26.1 vs. 23.3 kg/m^2^, *p* < 0.001) as well as greater waist circumference (86.9 vs. 79.0 cm, *p* < 0.001). The prevalence of obesity was also significantly higher among individuals with sarcopenia (54.1% vs. 25.9%, *p* = 0.003). The two groups showed no remarkable differences in terms of baseline demographics, nutritional parameters (energy and protein), and metabolic indicators (fasting glucose, total cholesterol, and vitamin D). Furthermore, the distribution of comorbidities, such as HTN, hyperlipidemia, OA, osteoporosis, and depression, was comparable between the two groups. Although not statistically significant, a lower proportion of individuals with sarcopenia met the WHO physical activity guidelines compared with those without sarcopenia (73.5% vs. 88.1%, *p* = 0.070).

### 3.2. Clinical Predictors of Sarcopenia in Patients with RA

Univariate analyses demonstrated that male sex (OR: 6.79; 95% CI: 2.76–16.67, *p* < 0.001) and obesity (OR: 3.37; 95% CI: 1.51–7.53, *p* = 0.003) were significantly associated with the presence of sarcopenia (Table 2). After adjusting for age, obesity, physical activity, and protein intake, both male sex (OR: 4.17; 95% CI: 1.48–11.77, *p* = 0.007) and obesity (OR: 3.06; 95% CI: 1.16–8.07, *p* = 0.024) persisted as independent predictors of sarcopenia. Meeting the WHO physical activity guideline showed a trend towards association (OR: 0.39; 95% CI: 0.14–1.06, *p* = 0.064), but did not demonstrate a significant effect. Neither age nor daily protein intake reached statistical significance regarding sarcopenia risk in either the univariate or multivariable analyses.

### 3.3. Sarcopenia Prevalence and Risk According to BMI Category

**Figure 1:**
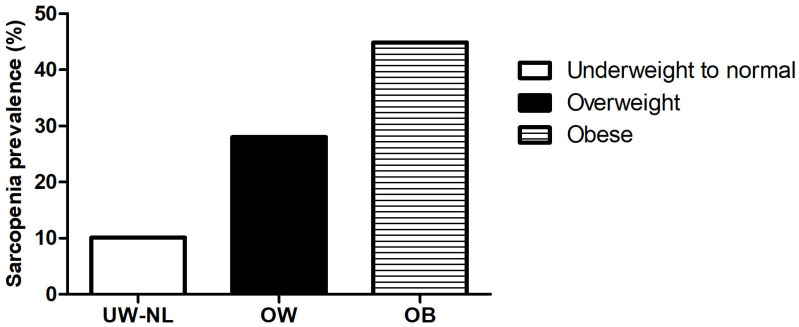
Prevalence of sarcopenia according to BMI categories in patients with RA.

Sarcopenia prevalence demonstrated a stepwise increase across BMI categories, rising from 10.1% in the underweight and normal BMI group (<18.5 and 18.5–22.9 kg/m^2^) to 28.0% in the overweight group (23.0–24.9 kg/m^2^) and further to 44.9% in the obese group (≥25.0 kg/m^2^) (Figure 1). In the multivariable logistic regression analysis adjusted for age and sex, the odds of sarcopenia were significantly higher in overweight (OR: 3.07; 95% CI: 1.02–9.22, *p* < 0.001) and obese (OR: 6.11; 95% CI: 2.06–18.12, *p* < 0.001) groups compared with the underweight and normal BMI group as the reference (Table 3). These findings support a dose–response association between increasing BMI and the likelihood of sarcopenia.

### 3.4. Sex-Specific Multivariable Analysis of Risk Factors for Sarcopenia

Sex-stratified multivariable analyses were conducted to examine potential differences in factors associated with sarcopenia between male and female participants (Table 4). In the male patients, not meeting the WHO physical activity guideline was markedly linked to a heightened risk of sarcopenia (OR: 13.00; 95% CI: 1.90–88.75, *p* = 0.012). Additionally, BMI was positively associated with sarcopenia in males (OR: 1.33; 95% CI: 1.05–1.68, *p* = 0.022), although age was not significantly related to sarcopenia (OR: 1.03; 95% CI: 0.99–1.08, *p* = 0.127). In contrast, among the female patients, only BMI was significantly associated with sarcopenia (OR: 1.35; 95% CI: 1.14–1.61, *p* = 0.001). Age and physical activity did not reach statistical significance in the female group (all *p* > 0.05). These results suggest that physical activity may influence the risk of sarcopenia differently by sex, although this finding should be interpreted with caution. Due to the limited number of participants in the sex-stratified analyses, particularly among men with sarcopenia, the estimates were associated with wide CIs.

## 4. Discussion

In this nationally representative cohort from the KNHANES, the weighted prevalence of sarcopenia in patients with RA was about 22.7%. This prevalence is substantially higher than that reported in the general Korean population and underscores the clinical burden of sarcopenia in individuals with RA [28]. Furthermore, our multivariable analyses identified male sex (OR: 4.17; 95% CI: 1.48–11.77) and obesity (OR: 3.06; 95% CI: 1.16–8.07) as independent risk factors for sarcopenia. The current findings support previous evidence that sarcopenia in RA is associated with sex and obesity [9,29]. In sex-stratified analyses, physical inactivity was significantly associated with sarcopenia only in male patients, whereas BMI remained a significant risk factor in both sexes. Our observations underscore the complex interaction between sex, body composition, and physical activity in RA-related sarcopenia. Furthermore, they underscore the clinical significance of this comorbidity, warranting individualized evaluation and management approaches in susceptible groups.

Several pathophysiological mechanisms can explain the association between RA and sarcopenia. Sarcopenia is a multifactorial condition involving age-related anabolic resistance, chronic inflammation, hormonal changes, physical inactivity, inadequate nutrition, and neuromuscular degeneration [30]. In patients with RA, the persistent release of systemic inflammatory mediators, such as TNF-α, IL-6, and IL-1β, disrupts muscle protein regulation by inhibiting protein synthesis via the insulin-like growth factor-1 (IGF-1)/Akt/mTOR signaling pathway and promoting proteolysis through the ubiquitin–proteasome system [10,31]. Additionally, chronic inflammation causes pain, joint stiffness, and fatigue, which collectively limit physical activity, exacerbate muscle atrophy, and contribute to functional decline [32]. These findings support the central role of inflammation-related molecular and behavioral mechanisms in the pathogenesis of sarcopenia in patients with RA [33]. Moreover, long-term use of glucocorticoids, which are frequently prescribed for the management of RA, may contribute to muscle wasting by increasing protein breakdown and impairing myogenesis. A previous study reported that glucocorticoid use was markedly associated with a higher likelihood of sarcopenia in patients with RA [29]. Therefore, these RA-specific factors establish a chronic catabolic state that promotes the development of sarcopenia.

These mechanisms may partially explain why male sex and excess weight are independently associated with sarcopenia in our analysis. While age-related declines in anabolic hormones like testosterone contribute to muscle loss, male patients in this study showed a significantly higher vulnerability to sarcopenia. This is notable because post-menopausal women experience a more rapid loss of estrogen, suggesting that sex-specific differences in RA involve factors beyond hormonal changes alone. Given that men generally have a greater baseline muscle mass, the catabolic effects of RA-associated chronic inflammation may result in muscle wasting that is more clinically significant than in women. A previous study also reported that age-related muscle loss progressed more rapidly in men than in women [34,35]. Furthermore, the regulation of muscle protein in response to inflammation differs by sex. Specifically, it has been suggested that the IGF-1/Akt/mTOR axis in men may be more susceptible to inhibition by inflammatory cytokines [36]. In addition to these biological factors, our analysis revealed that physical inactivity was significantly associated with sarcopenia only in male patients. This suggests that lifestyle adjustments in response to RA symptoms, specifically regarding physical activity, may play a critical role in maintaining muscle mass in an inflammatory state. Thus, the higher prevalence of sarcopenia in men likely results from a combination of greater absolute muscle loss, a higher biological sensitivity to inflammation, and a stronger link between physical inactivity and muscle depletion. These findings suggest that sex-specific hormonal and inflammatory profiles may significantly influence susceptibility to sarcopenia in patients with RA.

Data from this research demonstrated that the prevalence of sarcopenia increased progressively with BMI category, reaching 44.9% among patients with obesity, which suggests a dose–response relationship between increasing adiposity and the risk of muscle loss. While higher body weight is traditionally viewed as a protective mechanical load [37], our findings align with recent clinical observations identifying obesity as an independent risk factor for muscle depletion in RA [28,29]. This clinical discrepancy can be explained by the concept of sarcopenic obesity, which is characterized by the coexistence of excessive adiposity and reduced muscle mass [38]. In this condition, adipose tissue-derived proinflammatory adipokines, such as leptin and IL-6, impair muscle metabolism, while obesity-induced insulin resistance disrupts the anabolic signaling required for muscle protein synthesis [39]. Additionally, obesity-related physical limitations may further reduce the physical activity levels of patients with RA, thereby accelerating muscle loss. This issue is especially critical in Asian populations, including Korean cohorts [40,41]. These individuals often have a lower baseline of muscle mass, which makes them more vulnerable to sarcopenic obesity even at a relatively lower BMI. Thus, the concurrence of obesity and sarcopenia in RA patients is a critical clinical factor that can significantly worsen disease progression rather than it being a simple coincidence, as reduced muscle mass in these patients can severely impair physical function and increase disability while being masked by excess adiposity. This overlap makes disease management more difficult, as weight-based assessments frequently underestimate the severity of muscle loss. Consequently, identifying sarcopenic obesity in patients with RA may be important for optimizing treatment strategies, including targeted nutritional support and tailored exercise interventions. Our findings ultimately highlight the need for routine assessments of muscle mass and function in patients with RA, especially among men and those with obesity.

Beyond physical disability, the clinical importance of sarcopenic obesity in RA is closely linked to cardiometabolic comorbidities. The synergistic effect of adiposity and systemic inflammation not only reduces muscle mass but also promotes endothelial dysfunction and cardiovascular disease risk [3]. Sex-specific inflammatory responses further contribute to the cardiovascular risks associated with sarcopenic obesity. For instance, recent research on T2MI reveals that women may be more susceptible to a high inflammatory burden, as it is driven by an imbalance in myocardial oxygen supply and demand rather than acute plaque rupture [4]. Although our findings emphasize the high risk of sarcopenia in men, these observations suggest that the interaction between sex and inflammation in RA affects musculoskeletal and cardiovascular health in distinct ways. Consequently, our results underscore the importance of a comprehensive risk assessment that considers both body composition and sex-specific cardiovascular risks in patients with RA.

The present study has several limitations. First, due to its cross-sectional design, causality between the observed risk factors and sarcopenia cannot be determined. Second, RA diagnoses and comorbidities were based on self-reported physician diagnoses or current treatment status, which may be subject to recall bias. Moreover, although related musculoskeletal conditions (such as OA) are assessed separately in the KNHANES, some degree of misclassification cannot be completely ruled out, potentially influencing the estimated prevalence of sarcopenia. Third, physical activity was assessed using self-reported questionnaires rather than objective measures, which may have affected its accuracy. Furthermore, a key limitation of this study is the lack of detailed RA-specific clinical variables, such as disease activity, disease duration, serological markers, functional status, and information on pharmacological treatments. These factors are known to influence muscle mass and may contribute to the development of sarcopenia in patients with RA. Therefore, residual confounding cannot be excluded, and the observed associations should be interpreted with caution. Additionally, the sex-stratified analyses are limited by a constrained sample size, particularly men with sarcopenia (*n* = 18), which may have reduced statistical power and resulted in wide confidence intervals. Consequently, these findings should be interpreted as preliminary rather than definitive. Future studies incorporating detailed clinical data are needed to better elucidate these relationships. However, an important strength of this study is the use of a large, nationally representative dataset to examine weight-adjusted ASM criteria for sarcopenia in the Korean population.

## 5. Conclusions

In conclusion, our findings emphasize the substantial burden of sarcopenia among patients with RA, and identify male sex and obesity as important risk factors. Clinicians should consider routine assessments of muscle mass and function in RA management, especially in high-risk individuals. Future research using longitudinal data is warranted to clarify the causal mechanisms and evaluate interventions aimed at preventing sarcopenia in this patient population.

## Figures and Tables

**Table 1:** Baseline characteristics of study participants according to sarcopenia status.

Variables	Non-Sarcopenia (*n* = 194)	Sarcopenia (*n* = 44)	*p* Value
Age, years	56.0 (53.4–58.6)	52.2 (45.4–59.0)	0.279
Male, %	15.3 (9.7–23.2)	55.0 (37.0–71.8)	<0.001
BMI, kg/m^2^	23.3 (22.8–23.8)	26.1 (24.9–27.2)	<0.001
Waist circumference (cm)	79.0 (77.5–80.5)	86.9 (84.2–89.6)	<0.001
Current smoker, %	60.6 (43.5–75.5)	66.2 (45.8–82.0)	0.662
Current drinker, %	39.0 (30.7–48.0)	51.9 (33.7–69.6)	0.212
Obesity, %	25.9 (19.6–33.3)	54.1 (35.9–71.2)	0.003
Meets WHO physical activity guideline	88.1 (81.3–92.7)	73.5 (51.5–87.8)	0.070
EQ-5D index	0.9 (0.8–0.9)	0.8 (0.7–0.9)	0.448
Intake energy, g/day	1665.8 (1524.5–1807.0)	1642.4 (1408.7–1876.2)	0.868
Protein, g/kg/day	57.7 (51.4–64.1)	58.5 (45.7–71.3)	0.920
Fasting glucose	96.3 (91.5–101.2)	106.2 (86.8–125.6)	0.337
Total cholesterol	189.0 (183.5–194.6)	189.5 (173.4–205.6)	0.958
25 (OH) Vitamin D	18.5 (16.8–20.2)	17.3 (15.1–19.6)	0.401
Comorbidities, %			
Hypertension	28.0 (21.4–35.8)	24.3 (11.7–43.8)	0.680
Hyperlipidemia	14.1 (9.5–20.5)	20.2 (8.5–40.7)	0.428
Osteoarthritis	23.4 (16.6–31.8)	13.4 (5.5–29.1)	0.161
Osteoporosis	36.6 (28.6–45.5)	21.9 (9.9–41.8)	0.131
MDD	15.7 (10.2–23.4)	14.3 (5.2–33.6)	0.833

Values are presented as weighted means (95% confidence intervals) for continuous variables and as weighted percentages (95% confidence intervals) for categorical variables. *p*-values were calculated using a complex sample general linear model, accounting for sample weights, strata, and clusters. *n* represents an unweighted sample. All estimates are weighted to represent the Korean population. WHO, World Health Organization; MDD, major depressive disorder; EQ-5D, EuroQol-5-Dimension.

**Table 2:** Logistic regression analysis of factors associated with sarcopenia in patients with RA.

Variables	Univariate		Multivariate	
	OR (95% CI)	*p* Value	OR (95% CI)	*p* Value
Male	6.79 (2.76–16.67)	<0.001	4.17 (1.48–11.77)	0.007
Age	0.98 (0.96–1.01)	0.256	1.00 (0.96–1.03)	0.781
Obesity	3.37 (1.51–7.53)	0.003	3.06 (1.16–8.07)	0.024
Meets WHO physical activity guideline	0.37 (0.13–1.12)	0.078	0.39 (0.14–1.06)	0.064
Daily protein intake	1.00 (0.99–1.01)	0.919	1.00 (0.98–1.01)	0.597

Values are presented as weighted odds ratios (ORs) with 95% confidence intervals (CIs) for univariate and multivariate logistic regression models. Multivariate analysis included adjustments for age, sex, obesity, physical activity, and daily protein intake, along with sample weights, strata, and clusters based on a complex survey design. WHO, World Health Organization.

**Table 3:** Sarcopenia prevalence and odds ratios according to BMI category in patients with RA.

BMI Category (kg/m^2^)	Weighted *N*	Weighted Sarcopenia N	Weighted Prevalence (%)	OR (95% CI)	*p* Value
Underweight and Normal BMI (<18.5 and 18.5–22.9, respectively)	48,964,641	4,929,499	10.1	1.00 (Reference)	
Overweight (23.0–24.9)	18,109,720	5,072,295	28.0	3.07 (1.02–9.22)	<0.001
Obese (≥25.0)	24,395,840	10,947,704	44.9	6.11 (2.06–18.12)	<0.001

*N* and the percentages are weighted estimates that account for the complex survey design. BMI, body mass index; OR, odds ratio; CI, confidence interval. Weighted proportions may not sum to 100% due to rounding and the application of sampling weights. *p*-values were calculated using the underweight and normal BMI group as the reference.

**Table 4:** Multivariable logistic regression analysis of factors associated with sarcopenia in male and female patients with RA.

Variables	Male OR (95% CI), *p*	Female OR (95% CI), *p*
Age	1.03 (0.99–1.08), *p* = 0.127	1.00 (0.96–1.03), *p* = 0.786
BMI	1.33 (1.05–1.68), *p* = 0.022	1.35 (1.14–1.61), *p* = 0.001
Not meeting WHO physical activity guideline	13.00 (1.90–88.75), *p* = 0.012	0.59 (0.12–3.01), *p* = 0.520

Values are presented as odds ratios (ORs) with 95% confidence intervals (CIs). Separate multivariate logistic regression models were fitted for male and female patients, adjusting for age, body mass index (BMI), and adherence to the World Health Organization (WHO) physical activity guidelines. The analyses used a complex survey design that included sampling weights, strata, and clusters. The number of participants included in the sex-stratified analyses was 45 for men (18 with sarcopenia and 27 without) and 193 for women (26 with sarcopenia and 167 without).

## Data Availability

Data are available in a public repository. The datasets used in this study are available from the Korea National Health and Nutrition Examination Survey (KNHANES) website (https://knhanes.kdca.go.kr/knhanes/, accessed on 20 April 2026).
